# Supplementary material for: Correlated electronic states at domain walls of a Mott-charge-density-wave insulator 1T-TaS2
Source: Nat Commun. 2017 Aug 30;8:392. doi: 10.1038/s41467-017-00438-2 (PMC5577034; doi:10.1038/s41467-017-00438-2)
Supplement: Supplementary file 1 — Supplementary Information [file 41467_2017_438_MOESM1_ESM.pdf]

Description of Supplementary Files

File Name: Supplementary Information
Description: Supplementary Figures

File Name: Peer Review File

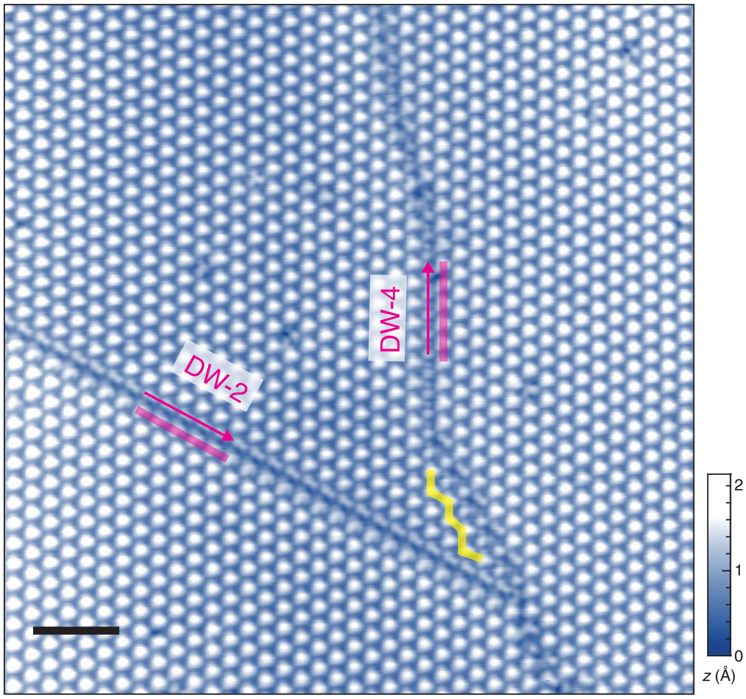

Supplementary Figure 1. **Domain walls in the commensurate CDW phase.** STM images of the insulating commensurate CDW domains ($I_t = 500$ pA, $V_s = -0.50$ V, $L^2 = 40 \times 40$ nm^2) including intrinsic domain walls. There are two types of edges, straight and zigzag zigzag ones. They are highlighted by magenta and yellow lines, respectively. The zigzag ones are rarely observed in the commensurate CDW phase. In the main text, we discuss the atomic and electronic structure of the domain walls with straight edges. Scale bar, 5 nm

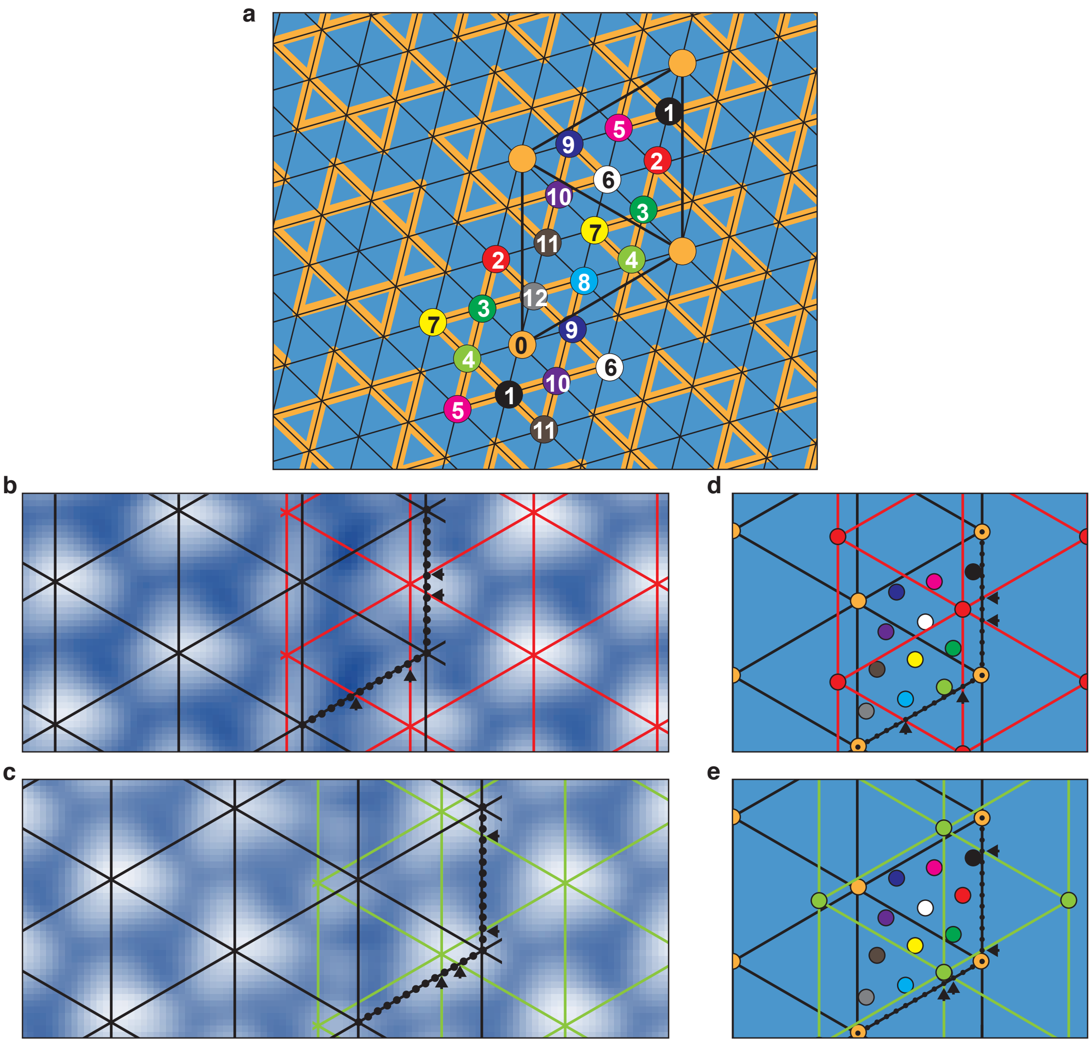

Supplementary Figure 2. **Identifications of anti-phase domain walls in the commensurate CDW phase.** **(a)** David-star superlattice. Each CDW unit includes 13 Ta atoms which marked by the color balls with numbers. **(b)** and **(c)** Two STM images of the domain walls shown in Fig. 2 of the main text. The triangular grids for each CDW domains are intersected at four points marked by black arrows on the unit cell of CDW superlattice. **(d)** and **(e)** The superimposed triangular grids shown in **b** and **c** with 13 Ta atoms in the CDW unit cell. They clearly show that the centers of David-stars are shifted from 0 to 2 and 4 Ta atoms across the domain wall, respectively.

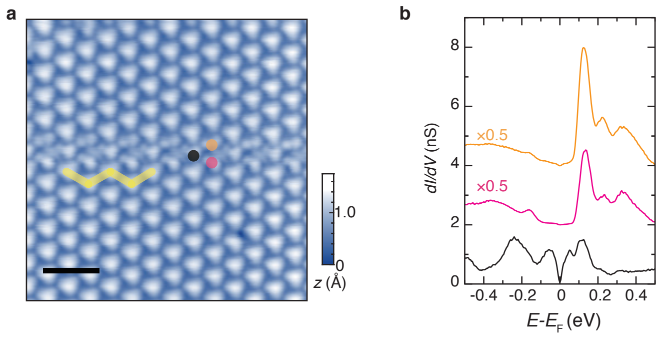

Supplementary Figure 3. **Localized in-gap states of the zigzag domain wall.** **(a)** STM images of the zigzag domain wall. The yellow line highlights the zigzag edge. Scale bar, 3 nm. **(b)** dI/dV spectra were acquired on the zigzag domain wall. The colors of the curves indicates the measurement sites marked in **a** as colored dots.

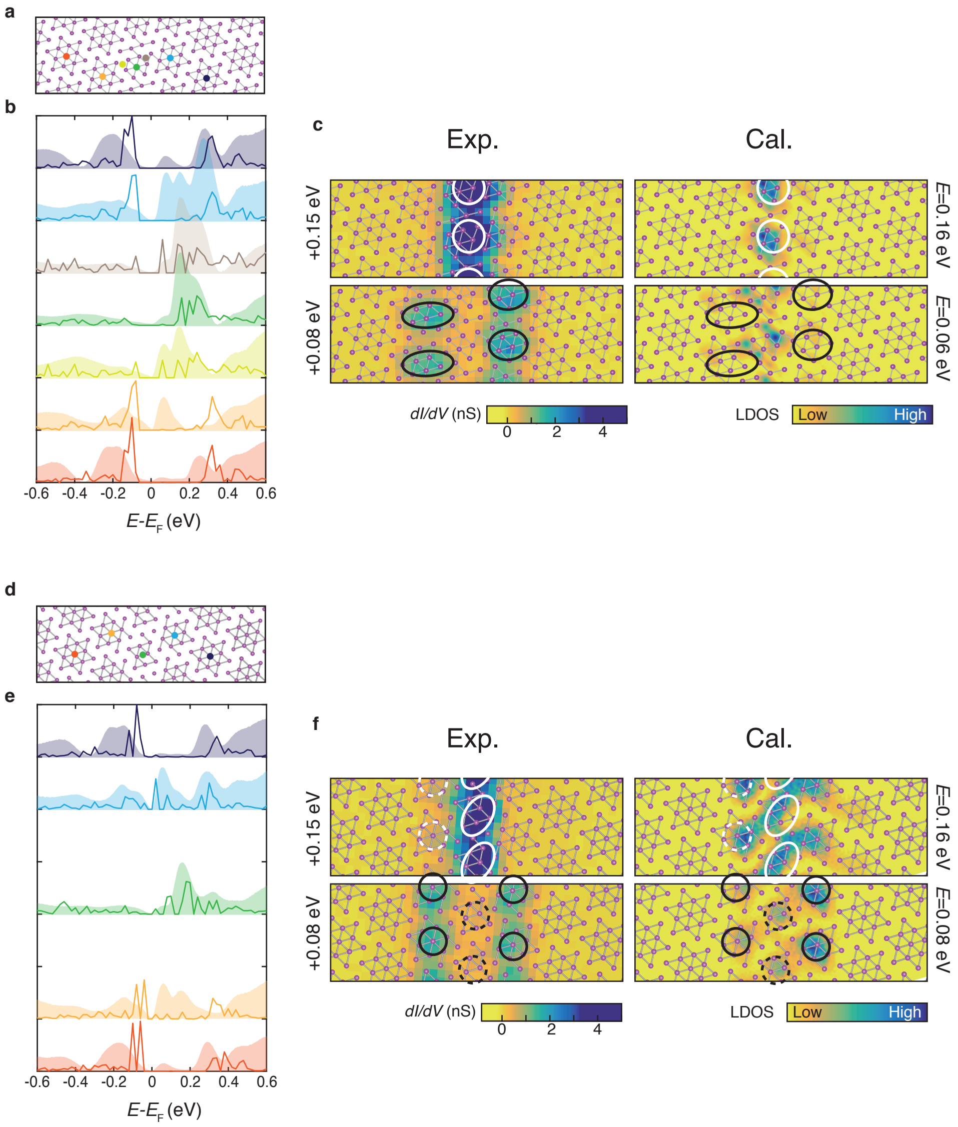

Supplementary Figure 4. **Direct comparisons between experimental and theoretical spectra.** **(a)** and **(d)** The calculated atomic structures of the $2^{\rm nd}$ and the $4^{\rm th}$ domain wall at U=2.50 eV. **(b)** and **(e)** The dI/dV curves (color filled ones) of the $2^{\rm nd}$ (**a**) and the $4^{\rm th}$ (**d**) domain wall are superimposed with the calculated LDOS spectra with gaussian broadening with a full width half maximum of 25 meV. The measured positions are marked by colored dots on the schematics for the reconstructed atomic configurations of the domain walls (**a** and **b**). All of dI/dV spectra are well consistent with the calculated LDOS ones except for some discrepancies in the spatial distribution of the edge states. **(c)** and **(f)** The spatial distribution of the in-gap states on the domain wall and edges. White and black circles indicate the domain wall and edge states, respectively. Dashed circles illustrate weak contrasts at the given energy levels. The charge modulations of domain wall and edge states have a same periodicity of the domain CDW reconstruction along the domain walls.

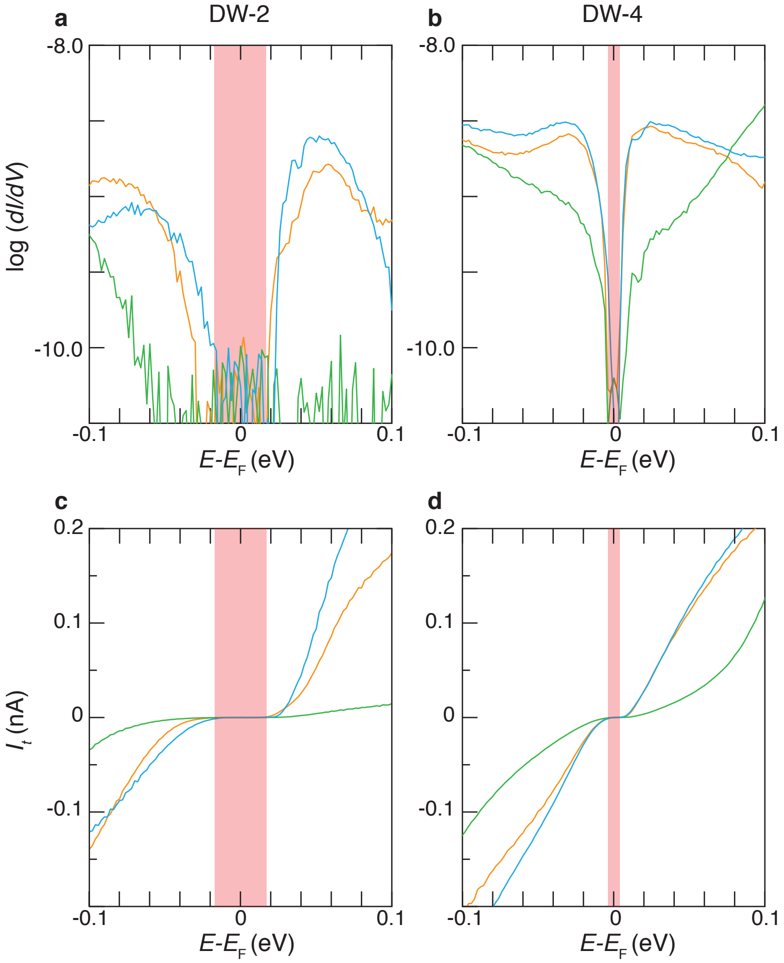

Supplementary Figure 5. **Non-metallic properties of the domain wall and edges.** **(a)** and **(b)** Logarithm of the high resolution dI/dV spectra at the domain walls and edges. The colors of each curves are same with original dI/dV curves shown in Fig.2 of the main text. **(c)** and **(d)** $I(V)$ curves acquired with dI/dV spectra (**a** and **b**). They have clear plateaus at E_{F}. The red vertical lines indicate the zero conductance region in the spectra.

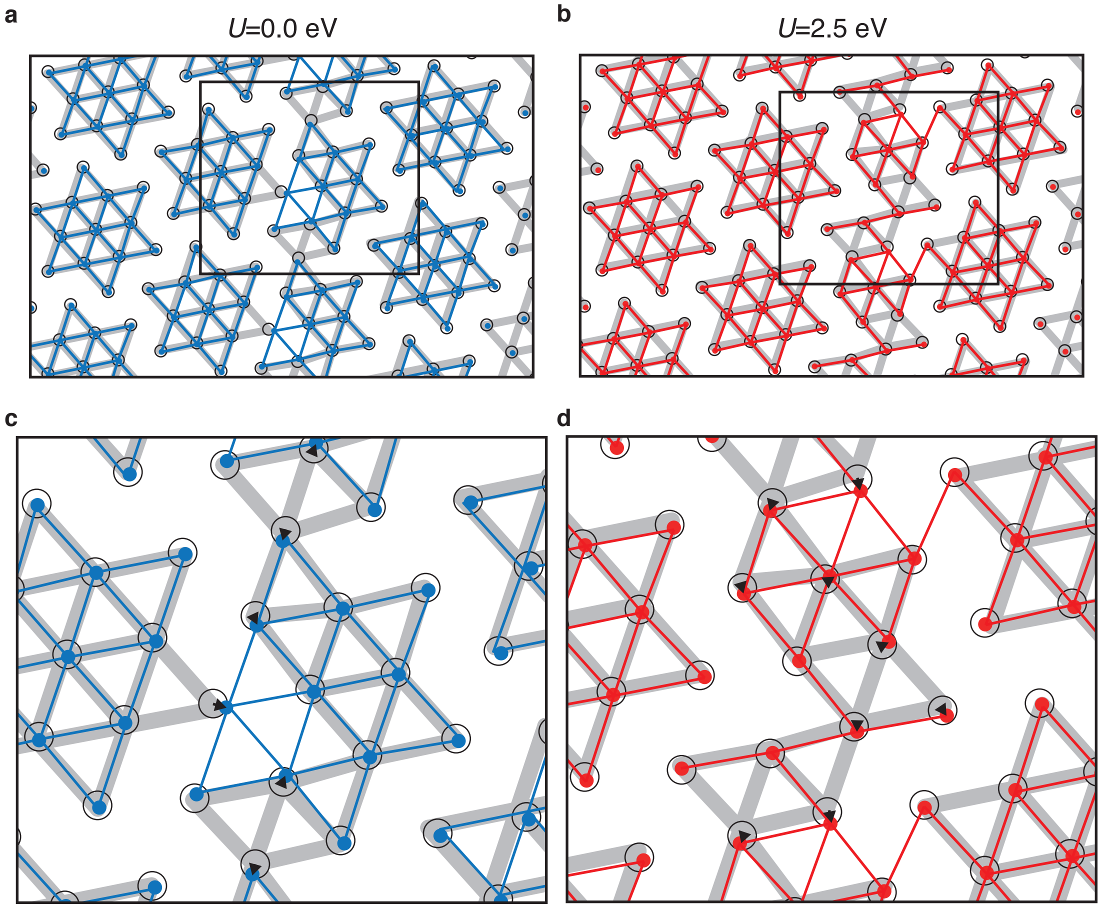

Supplementary Figure 6. Atomic displacements for domain wall and edge reconstructions. The empty circles and small dots indicates the initial and the relaxed atomic positions for the 2nd domain wall. The lines interconnecting the circles or the dots represent that atomic distances are shorter than the unit vector of $\times 1$ structure. The bold (thin) ones are for the initial (relaxed) structure. The atomic displacements are revealed by the non-concentric configurations between the circles and the dots. **(a)** For $U = 0.0$ eV, one of outer Ta atoms of David-star close to the domain wall move to the wall. As a result, the displacements lead to pairs of imperfect David-stars whose one corner is cut off. **(b)** For $U = 2.5$ eV, David-stars close to the domain wall tend to keep their reconstruction. This implies that correlation effect is strongly coupled to the CDW reconstruction. Inside the domain wall, Ta atom displacements lead to construct the alternative arrangement of the hexamers and the tetramers. **(c)** and **(d)** The enlarged schematics for the boxed regions in **a** and **b**. The atomic displacements are highlighted by arrows.

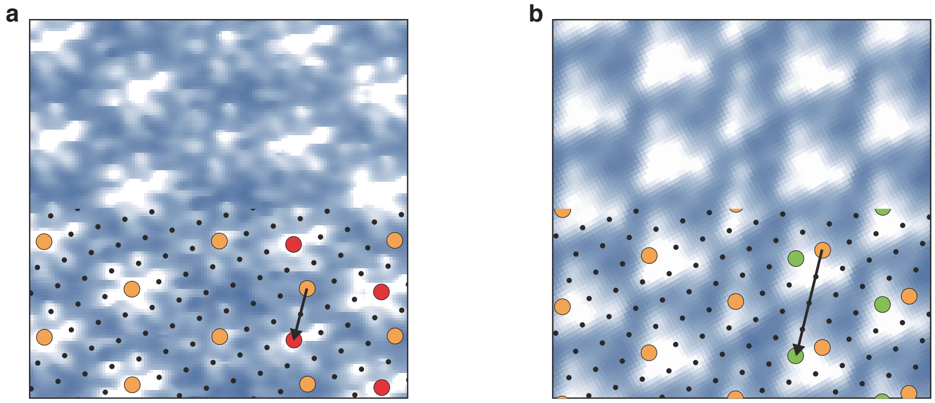

Supplementary Figure 7. **Atomic resolution STM images of the 2nd and 4th domain wall. (a)** STM image (V_s=-1.0 V, I_t=1.0 nA, and L^2 = 5×5 nm^2) of the 2nd domain wall. **(b)** STM image (V_s=-0.6 V, I_t=1.0 nA, and L^2 = 5×5 nm^2) of the 4th domain wall. The resolved atoms are highlighted by black dots. The CDW lattices are marked by larger dots whose colors identify the phase difference between the neighboring domains. The phase shifts across the domain walls are indicated by black arrows.
